# Electrical stimulation of Schwann cells on electrospun hyaluronic acid carbon nanotube fibers

**DOI:** 10.1371/journal.pone.0308207

**Published:** 2024-08-07

**Authors:** Judy Senanayake, Raymond R. Mattingly, Harini G. Sundararaghavan

**Affiliations:** 1 Department of Biomedical Engineering, Wayne State University, Detroit, MI, United States of America; 2 Department of Pharmacology and Toxicology, East Carolina University, Greenville, NC, United States of America; Changchun Institute of Applied Chemistry, Chinese Academy of Sciences, CHINA

## Abstract

Neurofibromatosis Type 1 (NF1) is a complex genetic disorder characterized by the development of benign neurofibromas, which can cause significant morbidity in affected individuals. While the molecular mechanisms underlying NF1 pathogenesis have been extensively studied, the development of effective therapeutic strategies remains a challenge. This paper presents the development and validation of a novel biomaterial testing model to enhance our understanding of NF1 pathophysiology, disease mechanisms and evaluate potential therapeutic interventions. Our long-term goal is to develop an invitro model of NF1 to evaluate drug targets. We have developed an *in vitro* system to test the cellular behavior of NF1 patient derived cells on electroconductive aligned nanofibrous biomaterials with electrical stimulatory cues. We hypothesized that cells cultured on electroconductive biomaterial will undergo morphological changes and variations in cell proliferation that could be further enhanced with the combination of exogenous electrical stimulation (ES). In this study, we developed electrospun Hyaluronic Acid–Carbon Nanotube (HA-CNT) nanofiber scaffolds to mimic the axon’s topographical and bioelectrical cues that influence neurofibroma growth and development. The cellular behavior was qualitatively and quantitively analyzed through immunofluorescent stains, Alamar blue assays and ELISA assays. Schwann cells from NF1 patients appear to have lost their ability to respond to electrical stimulation in the development and regeneration range, which was seen through changes in morphology, proliferation and NGF release. Without stimulation, the conductive material enhances NF1 SC behavior. Wild-type SC respond to electrical stimulation with increased cell proliferation and NGF release. Using this system, we can better understand the interaction between axons and SC that lead to tumor formation, homeostasis and regeneration.

## Introduction

Neurofibromatosis type 1(NF1) is a genetic condition characterized by peripheral nervous system tumors (PNSTs) including plexiform neurofibroma (pNFs) [1]. Plexiform Neurofibroma (pNF) is a pathological condition observed at birth in 20–25% NF1 patients [2] that is a result of genetic mutation of the NF1 gene leading to negative regulation of RAS signaling pathways [3, 4]. The NF1 gene encodes the protein neurofibromin which helps regulate cell growth. NF1 tumors initiate from Schwann cells (SC), which are support cells in the peripheral nervous system that help increase neuronal signal propagation. The NF1 Schwann cells (SC) with mutations in neurofibromin expression proliferate uncontrollably leading to Schwann cell tumors [5]. Loss of neurofibromin leads to tumors on nerve fibers (neurofibromas), which are often benign but can cause deformities, nerve compression and other complications. NF1 SCs recruit other cell types such as fibroblast, vascular endothelial cells and inflammatory cells\ [6, 7]. Increased drive/proliferation of NF1 SCdrives the formation of pNFs and cue the proliferation of fibroblasts and mast cells to form the tumor microenvironment [8, 9].

Plexiform neurofibromas are typically non-metastatic tumors with limited effective therapies for patients [10]. Recent clinical trials on drug therapy indicates successful outcomes with MAP kinase (MEK) inhibitors. Approximately ~70% of the children with inoperable pNF respond positively to MEK inhibitor treatments, while improving their quality of life [11]. The study conducted by Gross et al. evaluated the tumor shrinkage of a group of pNF subjects with inoperable tumors through orally administered MEK inhibitor selumetinib (AZD6244 or ARRY-142886). Approximately ~31% shrinkage of the tumor volume was observed as a response to the inhibitor drug by the study group [12]. However, this treatment causes side effects and cannot always be tolerated by the patient. Current studies on new therapies have been conducted in genetically modified mouse models [13], which have a high cost and only 10% of the tested therapies translate into successful applications in human trials [14]. Our long-term goal is to develop an organotypic three-dimensional in vitro model of NF1 that can be used to test drug therapies.

Most of the cell-based therapies for pNF are tested in 2D cultures, which does not sufficiently mimic the extracellular microenvironment [15] or animal models that are costly and may not mimic human cells [16]. We are developing a 3D cell culture system using electrospun hyaluronic acid (HA) nanofibers combined with carbon nanotubes (CNTs). We propose using electrically conductive nanofibers to simulate the neuronal component in our organotypic models. Electrospun HA scaffolds are developed to mimic properties of the axons on which the SC grow including mechanical, topographical and electrical cues. Using this system, we can study SC: Axon interactions. In this paper, we are evaluating the effect of electrical stimulation on Schwann cells as a first step towards this goal towards the organotypic model. Our previous research on utilizing HA-CNT fibers with chick derived DRG neurons developed a tissue-engineered platform to successfully test cellular behavior in the presence of multiple cues [17]. We are interested in how nanofibers cues that mimic axons effect the behavior or NF1+/+ and NF1-/- Schwann cells to study the mechanism behind SC proliferation and maturation.

In nerve regeneration applications, electrical stimulation within a tested safe range could lead to molecular cascades that support the electrical activity of the neuronal cells thereby leading to restoration of neuronal connections [18]. Electrical stimulation has been shown to release and enhance the secretion of a variety of growth-related proteins [19] such as interleukin-8 and FGF-1 from fibroblasts [20], BDNF from hippocampus neurons [21], and type II collagen from chondrocytes [22]. Multiple studies have indicated that SCs express neurotrophic factors at the injury site after electrical stimulation thereby leading to favorable microenvironment for regeneration [18]. During embryogenesis and regeneration, it is known that endogenous (21–140 mV/mm) electrical fields are present [23]. One previous study investigated the effect of electrical stimulation on neuropathic pain in NF1 patients [24]. However, the effect of electrical stimulation on cells in the pNF microenvironment is unknown and the effect of physiologically relevant electrical stimulation on pNF SC has not previously been studied.

In this study, we have developed a HA-CNT nanofibrous scaffold and custom stimulation set up to evaluate the behavior of wild-type and NF1 SC under electrical stimulatory conditions. We have quantified SC attachment, cell spreading/elongation and proliferation with and without stimulation. We have also evaluated NGF release from SC with and without electrical stimulation. Together, these results show that NF SC elongate, proliferate and release NGF on conductive nanofibrous scaffolds. In the presence of stimulation, we see that these properties decrease for NF SC but increase for WT SC. We conclude that WT SC proliferate and elongate under regenerative electrical stimulation conditions, while NF SC proliferate and elongate on conductive materials under low/no electrical conditions.

## Methods

### HA hydrogels

Hyaluronic acid (HA) hydrogels were made using previously described methods using 3% methacrylated HA and 0.05% I2959 photoinitiator [25]. Briefly, hydrogels were prepared by mixing 3% methacrylated HA (~30% methacrylated, tested with NMR, S1 Fig in S1 File), 1x PBS and 0.05% irgacure and UV crosslinked for 30 minutes.

### Electrospinning HA and HA-CNT aligned nanofibers

Nanofibers were prepared from the methods adopted from Steel et. al. [17]. Briefly, the fiber spin solution contains 2% methacrylated hyaluronic acid (HA), 2% polyethylene oxide (PEO, 900kDa), 0.05% I2959 photo-initiator dissolved in deionized water for controls (HA) and 0.01% multi walled carbon nanotubes (MWCNT) mixed in saline (HA-CNT). Aligned HA and 0.01% HA-CNT fibers were prepared by electrospinning on a rotating mandrel with a dispense rate (flowrate) of 1.7mL/h and a voltage of 17kV from a blunt 18 gauge needle, 11 cm from a rotating mandrel. All experiments used aligned fibers of HA and HA-CNT spun on methacrylated coverslips (round and square). All fiber samples were UV crosslinked for 30 minutes prior to hydration for experiments.

### Nanofiber characterization

Fibers were characterized using SEM imaging. The samples were placed on a sample holder (6 sample holder) and gold sputtered for 30 seconds. Gold coated samples were loaded into the SEM chamber and vacuum purged. The chamber pressure was below 10^−4^ *Torr* with extraction voltage set to 6 kV. The distance between the sample and bottom of the lens was maintained at 8 mm (Z axis) plus offset (manually performed for each sample). All adjustments for imaging (fine tuning) were made during slow focus to obtain clear images. Images were captured at 10k and 15k magnifications.

Fiber diameter and angle were measured using ImageJ (v1.53a) with at least 200 fibers per fiber type. Fiber diameter was measured manually. The angle tool was used to measure the orientation of the fibers. The bottom border was used as the reference 0° plane and all fibers projecting from this reference plane was measured. We calculated the percent of aligned fibers by dividing the among of fibers that deviated less than 20° from the neutral axis [26].

### Schwann cell culture

Immortalized NF1 patient derived Schwann cell (NF SC, NF-/-) line, ipNF 95.11b and Wild type Schwann cell (WT SC, NF+/+) line, ipn 02.8 were utilized for all experiments [4]. The cells were extracted from human patients diagnosed with Plexiform Neurofibroma in peripheral nerves. WT cells are non-tumor nerves while NF cells extracted from tumor sites in peripheral nerve [27]. All cells were cultured in DMEM (Gibco by Life Technologies) with 10% Fetal Bovine Serum (Hyclone by Life Science) and sub-cultured using 0.05% Trypsin (Gibco by Life Technologies).

### Attachment of Schwann cells

HA and HA-CNT fibers spun on (12 mm diameter) round coverslips were utilized for attachment experiments. The fibers were tested with and without a collagen coating (Corning by Life science/ Advanced BioMatrix). In this study, we used aligned nanofibers to mimic neurons in the neurofibroma. Our previous work investigated aligned and random fibers for various cell types [17, 26, 28–32]. The fibers were hydrated in 1x PBS for 48 hours before experiments. All four experimental fiber groups (HA and HA-CNT, with and without collagen) were seeded with 20,000 wild-type Schwann cells (WT SC) and NF SC per well and incubated overnight at 5% CO_2_ and 37°C. The cell seeded coverslips were moved to fresh wells 24 hours later for attachment and cell morphology was analyzed through fluorescence staining.

### Immunofluorescence staining

Samples were fixed using 4% Paraformaldehyde (PFA) and rinsed thrice with warm 1x PBS. The samples are permeabilized by 0.1% Triton X-100 in 1x PBS with FBS (wash buffer) and blocked in 10% goat serum for 1 hour. Schwann cells were incubated with antibody against s-100b (Sigma) as the primary overnight at 1:400 ratio, treated with anti-rabbit IgG as secondary (Alexa Fluro 594, Invitrogen) for 2 hours at 1:1000 ratio, and counterstained with DAPI (Fisher Scientific) for 5 minutes at 1:2500 ratio for nuclei staining. Wash buffer was used as the diluent for all staining components (dyes). The wells were washed thrice in between all staining steps with wash buffer and placed in 500 μl (attachment experiment) to 1000 μl (stimulation experiment) of 1x PBS protected from light until imaging. Stained samples were imaged using inverted Nikon microscope [Eclipse T*i*-E T*i*-E/B] with LED light source. Texas Red [s100b] and DAPI [cell nuclei] filters with 200-300ms exposure times were used for the imaging purposes. The images were analyzed using NIH ImageJ (version 1.53a) software. Aspect ratio, which is the longest length of the cell divided by the shortest length of the cell, and cellular spread area, which is the total area of the cell, were measured and statistically analyzed. Conditions were run in triplicate with at least 200 cells measured for each condition.

### Alamar blue assay

Alamar blue assays were conducted at 24, 48 and 72 hours on unstimulated samples to quantify the cellular proliferation on HA, HA-CNT and hydrogels. We also ran Alamar blue cell proliferation assays on stimulated HA-CNT nanofibers 48hrs post electrical stimulation. Briefly, Alamar blue solution was prepared at 10% of the well volume using cell culture media. Each well was treated with 500 μl of Alamar blue solution at the timepoints as indicated in the experiment. The plates were incubated protected from light for 6–7 hours to detect sufficient color gradient. A standard curve was used to calculate cell densities on experimental wells. The samples were pipeetted from the treated plates in triplicates of 100 μl and transferred into a 96-well plate, and read using Thermo Scientific Multiskan GO at 570 and 600nm to obtain the absorption gradient. The data was analyzed in terms of percentage reduction corresponding to the two wave lengths using a standard curve. Table 1 shows that percentage increase in cell number from 24 hours to 72 hours for all conditions. Percentage increase was calculated by the ratio of the change in cell density between 72 hours and 24 hours post seeding by the final cell density (72 hours). All samples were run in triplicate.

**Table 1:** Proliferation. Percentage increase in cell number from 24 hours to 72 hours for all conditions.

Experiment Type	Mode	NF Increment %	WT Increment %
**Stimulated Experiment**	**Unstimulated**	**64.3%±5.5**	**48.9%±20**
	**100mV/mm**	**36.4%±16**	**73.9%±5.0**
	**200mV/mm**	**44.0%±11**	**60.9%±11**
**Material Experiment**	**HA 24H-72H**	**24.0%±18**	**52.2%±7.5**
	**HACNT 24H-72H**	**29.0%±16**	**50.6%±15**

### Schwann cell stimulation

**Fig 1:**
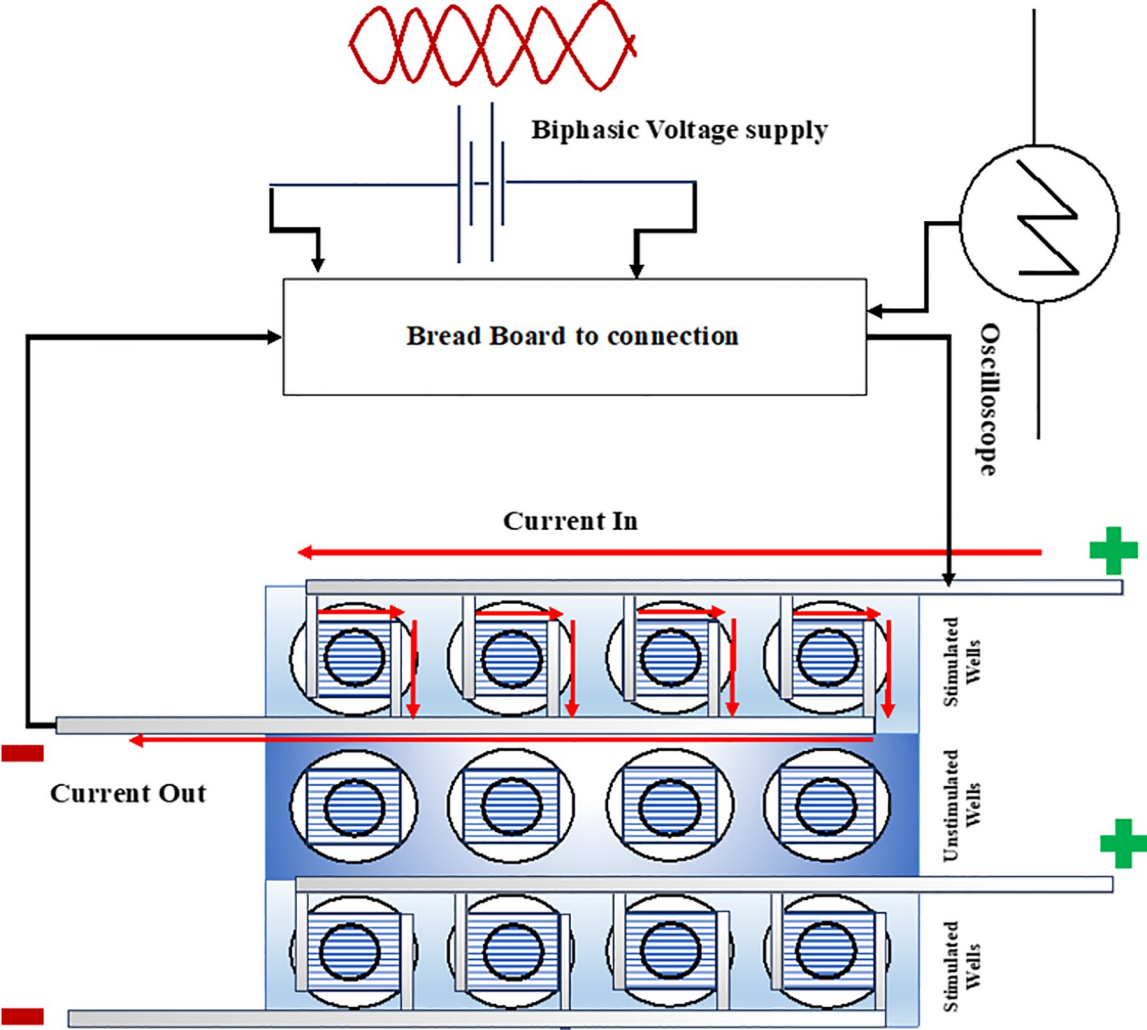
Schematic of the custom electrical stimulation setup. The stimulation wells are drilled within a 12-well plate. Two stimulation rows have 4-wells in series. The middle row of the 12-12ll plate is used the unstimulated control.

Custom stimulation well plates were prepared using 12-well tissue culture plates as previously described [17] (Fig 1). Briefly, custom electrical stimulation chambers were fabricated from 12- well cell culture plates by drilling a hole using a 6-mm bit on a Dremel drill press and sandwiching: glass coverslip, electrospun nanofibers, copper tape, Very High Bond tape (3M), and a custom cut well within a standard culture well (Fig 1). Copper electrodes were not in contact with the culturing medium; they are only in contact with the nanofibers. A Function Generator connected to a breadboard with 2 binding posts, 7.25″ × 7.5″ (Jameco) was used as the power source. The applied biphasic stimulus (Voltage In) and the stimulus after it passed through the stimulation chamber (Voltage Out) were captured from Channel 1 and Channel 2 of an oscilloscope (Rigol) connected to a Raspberry Pi running a Python script to store the data in near real time through the USB port connection. The plates were UV sterilized before the hydration to prevent contamination. The inner well of the stimulation plate is approximately 32 mm^2^. The fibers were hydrated using 1x PBS, and coated with collagen (Rat-tail Col1, Advanced BioMatrix). Each well was treated with 50 μl of collagen in acetic acid and left under the laminar flow hood for the acetic acid to evaporate and leave an adsorbed layer of collagen. WT and NF SCs were prepared, and each well was seeded with a cell density of 2,000 cells in 50μl of media initially to incubate the plates for attachment. The incubated plates were treated with 1ml of warm media after 2 hours and left incubated overnight. Before stimulation the media was exchanged to remove any floating cells.

The wells were stimulated at 20Hz biphasic square wave of 100 and 200mV/mm for 30mins. Unstimulated control group were also exposed to the same conditions during the stimulation of the experimental groups. The custom well plates were incubated for 48 hours, and the supernatants were collected at different three time points: immediately after stimulation (0 time point), 24 hours post stimulation and 48 hours post stimulation. Nerve growth factor (NGF) expression was measured through and ELISA assay. Controls were run with unstimulated wells that were maintained under the same experimental conditions. Alamar blue assay was run 48 hours after stimulation. Conditions were run in triplicated with four samples per trial (n = 12). Immunohistochemical staining was conducted at 48 hours post-stimulation. Conditions run in triplicate with n = 200 cells.

### NGF ELISA

ELISA was used to calculate the concentration of NGF release by stimulated SC in the media as previously described [26]. The supernatants extracted at different time points (immediately after stimulation, 24 hours and 48 hours) post electrical stimulation were tested for NGF release and compared to a standard curve. Standard curves were prepared with known concentrations of NGF dissolved in DMEM. A linear regression was run on the standard curve and used to calculate concentration of NGF in the supernatant.

### Statistics

Results were analyzed using ANOVA with Tukey’s posthoc tests for all experiments. Significance was set at p<0.01.

## Results

### Nanofibers

**Fig 2:**
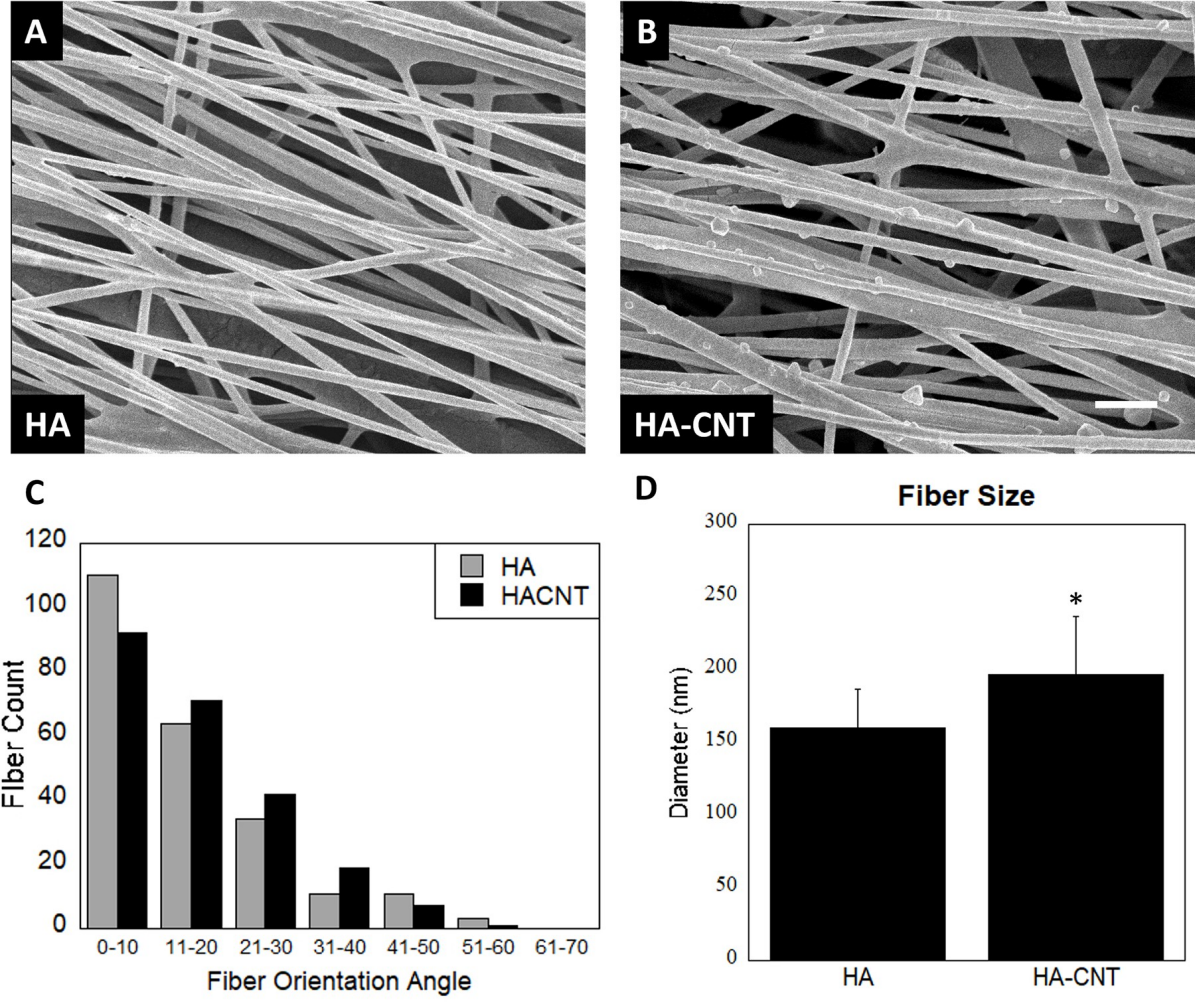
Fiber morphology: A and B show SEM images of HA and HA-CNT fibers. We can see bumps along the HA-CNT fibers which are formed from the MWCNTs within the fibers (See S 2 for TEM). SB = 1μm. C shows fiber orientation of electrospun nanofibers for both HA and HA-CNT, both of which are aligned. D shows fiber diameter of the two fiber types. (n = 200, p<0.01).

We analyzed at least 200 nanofibers with 10 SEM images for each fiber type (Fig 2A and 2B). The percentage of aligned fibers in each of the nanofiber groups HA and HA-CNT were 69% and 72% respectively (Fig 2C). Fiber diameter for HA and HA-CNT was 160±27nm and 197±40 nm respectively, with HA-CNT fibers significantly larger than HA fibers (Fig 2D). See S2 Fig in S1 File for TEM image of CNT within the HA nanofiber.

### Cell attachment and proliferation

**Fig 3:**
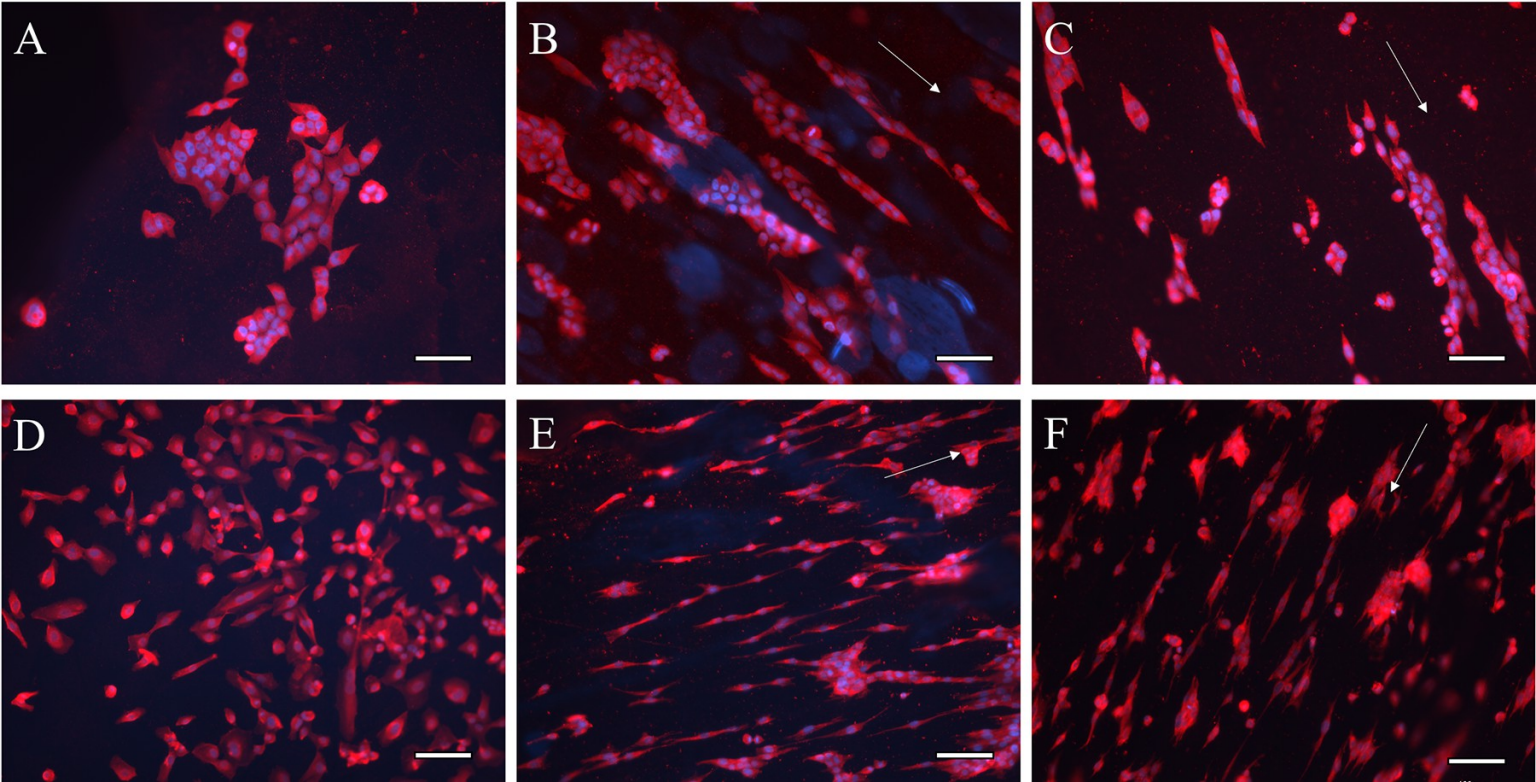
WT SC and NF SC morphology on HA hydrogels (A,D), HA nanofibers (B,E) and HA-CNT nanofibers (C,F). WT SC are rounder and tend to cluster (A,B,C), NF SC are more elongated (D,E,F). SB = 100 μm, S100 (Red), DAPI (Blue). Fiber alignment direction is indicated by white arrow in B,C,E,F. Hydrogels (A,D) do not have a topographical cue.

**Fig 4:**
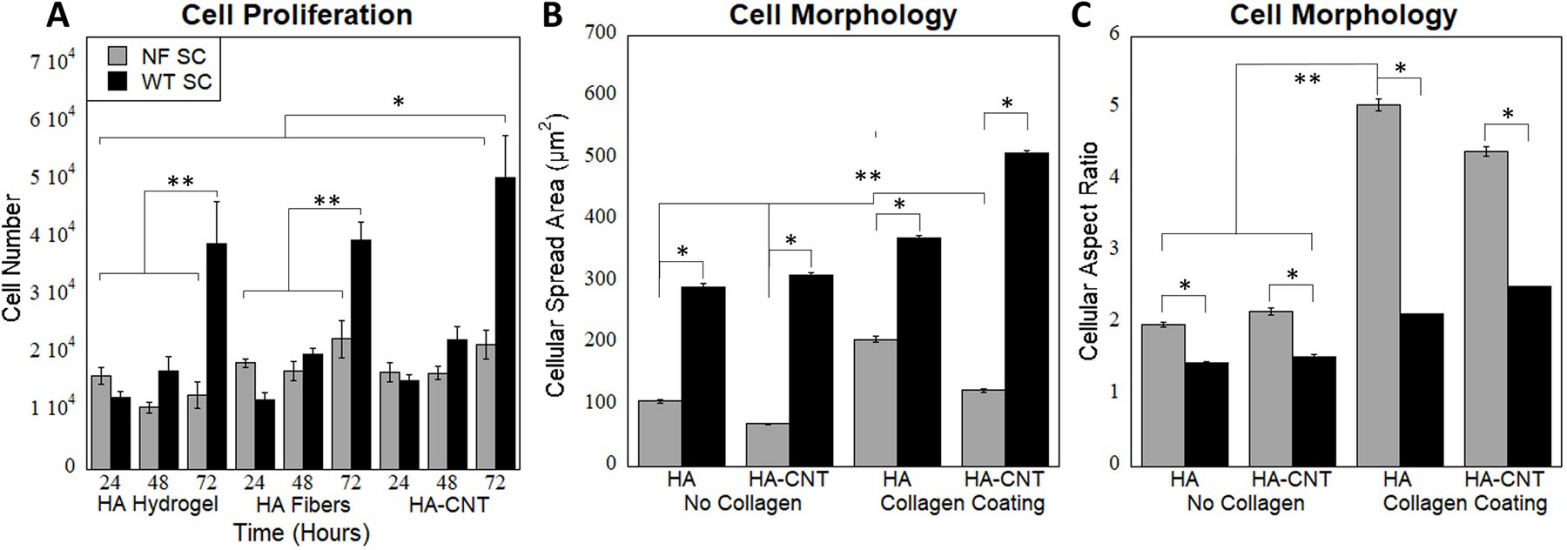
(A) Cell proliferation of NF SC and WT SC on hydrogels, HA Fibers and HA-CT Fibers. Cell proliferation was measured with Alamar blue and show increases with time. WT SC on HA-CNT fibers show significantly higher proliferation than all other conditions (*). WT SC at 72 hours was significantly higher than other conditions in their material group (**). Conditions run in triplicated with four samples per trial (n = 12) (B) Cell spread area with and without collagen coating. WT SC on HA-CNT has significantly higher cell spread area than all conditions. NF SC on HA has significantly higher cell spread area than all other NF SC conditions (**). WT SC has significantly higher cell spread area than corresponding NF SC (*). Conditions run in triplicated with 200 total cells per condition (n = 200) (C) Aspect ratio increases with collagen coating. NF SC has a higher aspect ratio than WT SC. NF SC on HA collagen has significantly higher aspect ratio than no collagen conditions (**). NF SC aspect ratio is significantly higher than the corresponding WT SC conditions (*). Conditions run in triplicated with 200 total cells per condition (n = 200). (p<0.01).

HA and HA-CNT nanofibers with and without collagen coating were evaluated for their effects on cellular aspect ratio and cellular spread area. Cellular images are shown in S3 Fig in S1 File. Fig 3 shows cells on HA hydrogels and HA nanofibers with a collagen coating. NF SC appear more aligned than WT SC in all conditions. WT SC are more rounded and appear to group together. These results are quantified in Fig 4.

We measured cell proliferation on HA hydrogels and collagen coated fibers and found that WT SC showed the most proliferation at 72 hours (Fig 4A). Cellular proliferation was measured using an Alamar blue assay at 24, 48 and 72 hours post cell seeding. WT SC showed the most proliferation in all material conditions (Fig 4A). Cell proliferation was higher for both cell types on fiber conditions compared to hydrogels. Percent increase in proliferation is quantified in Table 1.

Cellular spread area is a measurement of total area of the cell. The cellular spread area was found to be greater in WT cells than NF cells as indicated in Fig 4B. Spread area was greatest for WT cells on HA-CNT with collagen coating. Increased cellular area could correlate with an increase in roundness. Both WT and NF SCs showed increased elongation on collagen-coated fibers, however NF SC showed significantly more elongation compared with WT cells. Elongation was measured by aspect ratio in Fig 4C. NF SC on collagen coated HA nanofibers has a significantly higher aspect ratio than WT SC in all conditions.

### Electrical stimulation of Schwann cells

**Fig 5:**
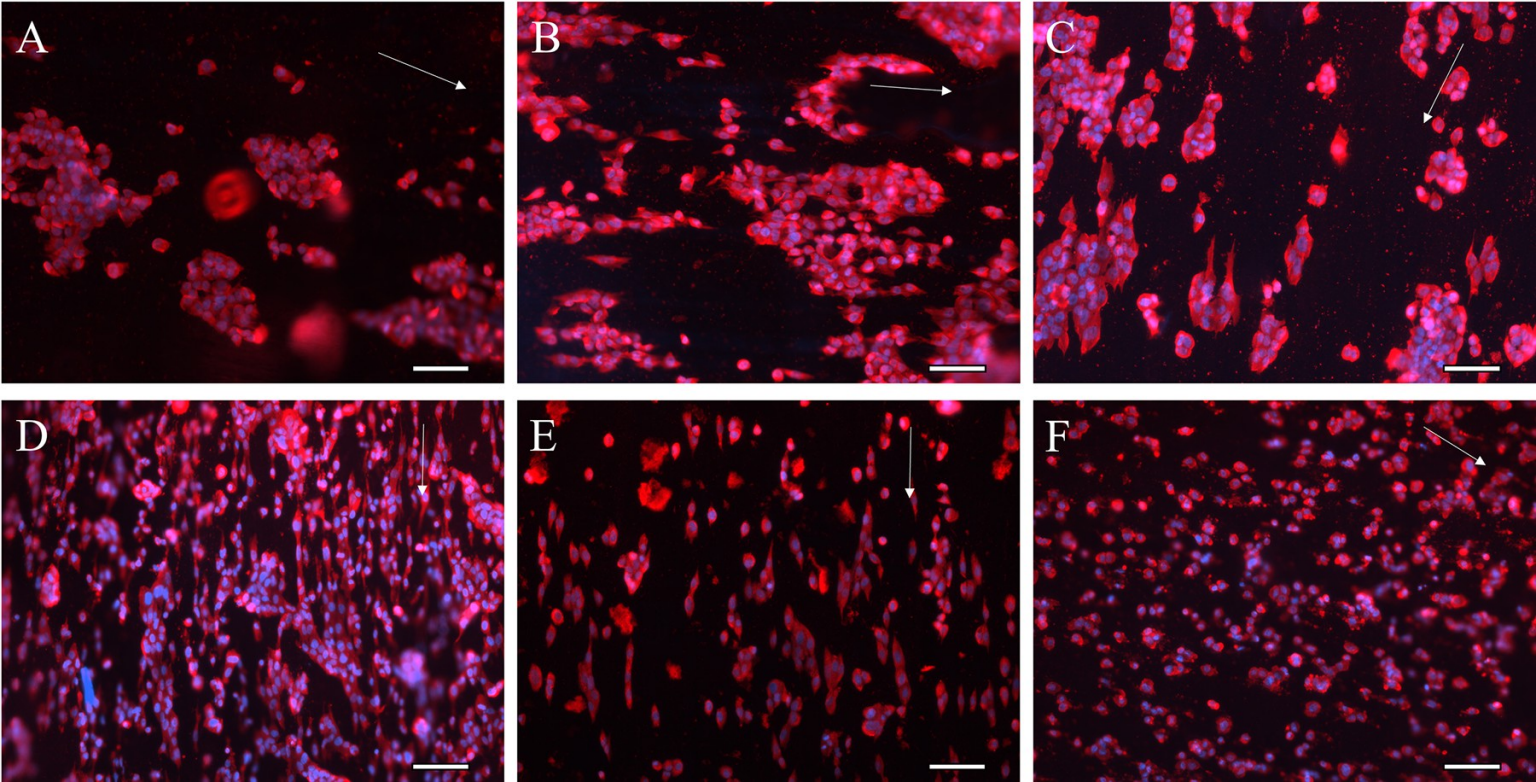
WT SC (A,B,C) and NF SC (D,E,F) morphology in unstimulated (A,D), and stimulated at 100 mV/mm (B,E) and 200 mV/mm (C,F). Immunohistochemical staining was done 48 hours post stimulation with S100 (red) and DAPI (blue). SB = 50μm. Fiber alignment is indicated by white arrow.

**Fig 6:**
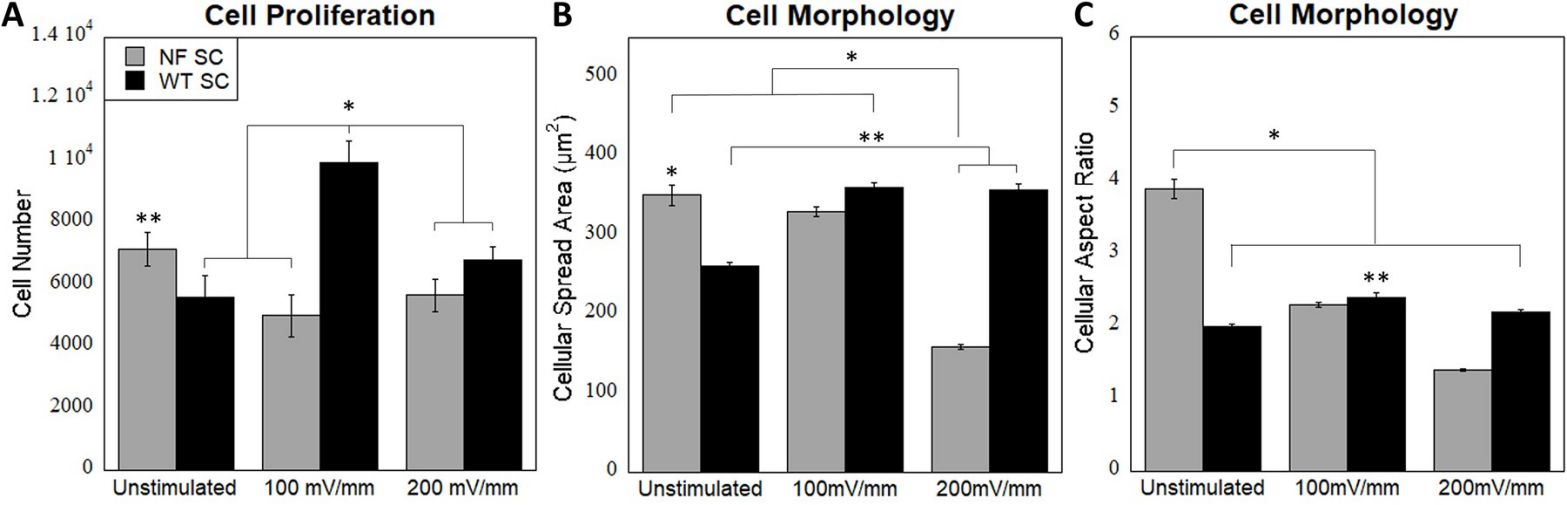
(A) Cell proliferation of NF SC and WT SC post electrical stimulation at 0, 100 and 200 mV/mm on HA-CNT nanofibers. Cell proliferation in WT cells increases post 100 mV/mm stimulation. WT SC cell number is significantly higher than other conditions except the unstimulated NF SCs (*). Unstimulated NF SC cell number is significantly higher than all other NF SC conditions (**). Conditions run in triplicated with four samples per trial (n = 12). (B) Cell spread area (total area of the cell) post electrical stimulation shows significantly decreased area for NF SC following 200 mV/mm stimulation and unstimulated WT SC (*). WT SCs stimulated at 200 mV/mm were significantly higher than NF SCs at 200 and unstimulated WT SCs (**). Conditions run in triplicate with n = 200 cells. (C) Aspect ratio (the ratio of the longest length of the cell to shortest) decreases with stimulation for both cell types. Unstimulated NF SCs were significantly higher than all other conditions (*). WT SCs stimulated at 100 mV/mm were higher than other conditions for WT SCs (**). Conditions run in triplicate with n = 200 cells. p<0.01.

We have previously investigated the effect of 30 mins of electrical stimulation at both 100 and 200 mV/mm voltage [17]. We used the same stimulation parameters in this study. Cell morphology of WT and NF SC with and without stimulation is shown in Fig 5. Additional cell images are available in S4 Fig in S1 File. WT SC cultured on nanofibers show clustering and rounder morphology with some elongation under 100 mV/mm stimulation (Fig 5A–5C). NF SC are more elongated with some rounding following stimulation at 200 mV/mm (Fig 5D–5F). At the stimulation parameters that we tested, cellular spread area is higher for WT cells than NF SC. Aspect ratio was highest of unstimulated NF SC (Fig 6B, 6C).

Following electrical stimulation, proliferation was measured for WT and NF cells using an Alamar Blue assay. The cells were cultured for 48hrs post electrical stimulation for 30mins. Cellular proliferation was highest for WT SC at 100 mV/mm stimulation. NF SC showed highest proliferation when unstimulated (Fig 6).

#### Electrical stimulation of Schwann cells releases Neural Growth Factor (NGF)

**Fig 7:**
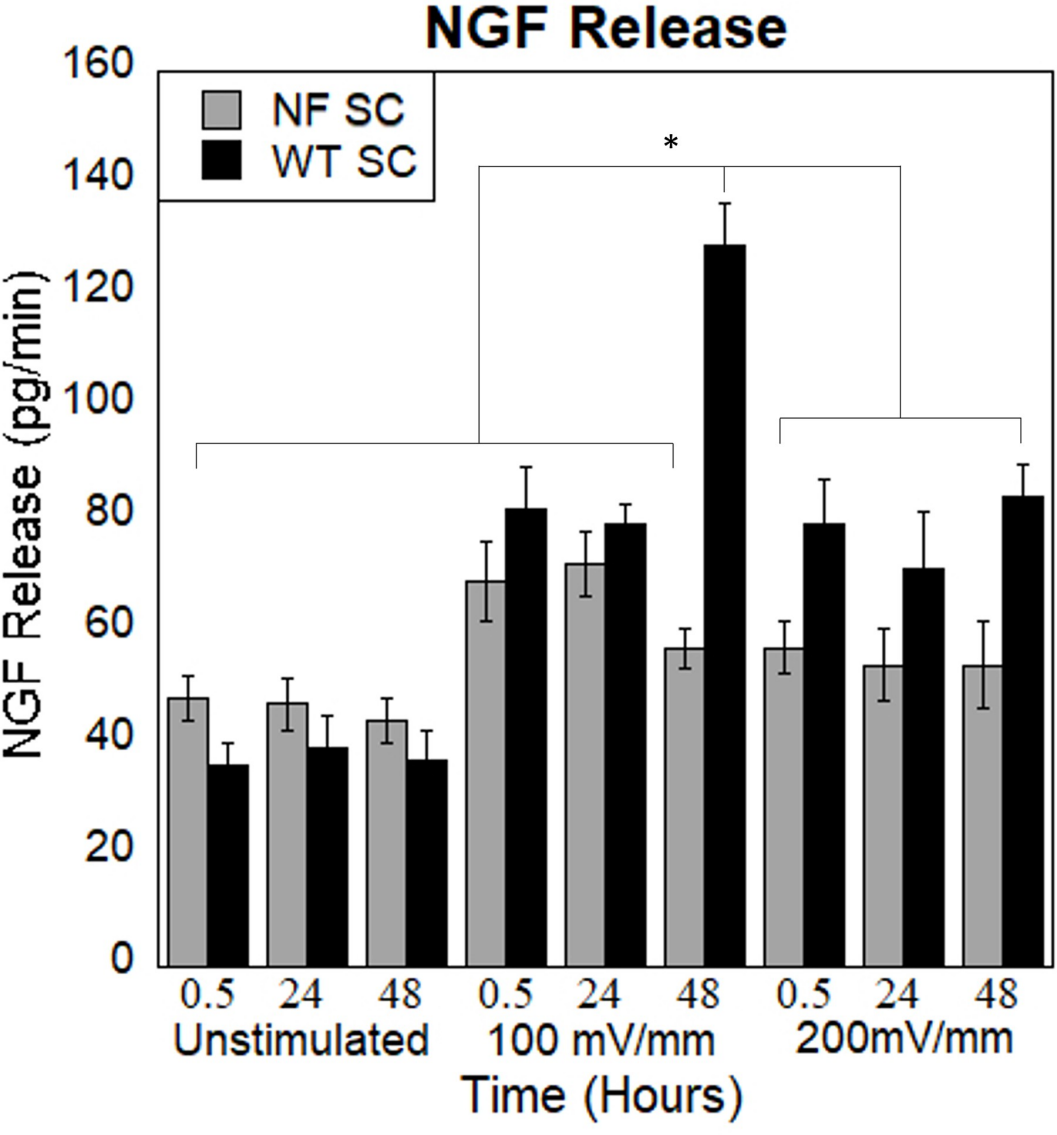
NGF release from SC with and without stimulation on HA-CNT nanofibers. Cells were cultured on HA-CNT scaffolds without stimulation and at 100 and 200 mV/mm stimulation. We see increased NGF release over time. WT SC at 48 hours, 100 mV/mm is significantly higher than all other conditions (*). (p<0.05).

NGF ELISA was conducted on the SC media to measure the amount of growth factor release following stimulation. A standard curve was developed to calculate the concentration of NGF release of the experimental groups. The values of NGF release for the three different time points (immediately after ES, 24h and 48h after ES) are shown on Fig 7. Highest NGF release was seen following 100 mV/mm for WT SC. NGF release increased for both cell types under 100 mV/mm stimulation.

## Discussion

In this study we demonstrated the cellular response of wild type Schwann cells (WT SC) and plexiform neurofibroma Schwann cells (NF SC) to electrical stimulation on an electrically conductive nanofiber biomaterial. The material system was designed with aligned hyaluronic acid fibers and ultra-low concentrations of multiwalled carbon nanotubes (HA-CNT) to mimic the physiological architecture of neurons while minimizing immune response. Our tunable system allows us to include additional neuronal cues to SC including topographical, adhesive and chemical cues in future studies. Controls were run with HA without CNTs, without stimulation and HA hydrogels that do not provide topographical cues.

Our previously published paper with HA-CNT nanofibers reported mechanics and surface contact angle properties [17]. We found that adding CNTs slightly decreased contact angle (HA: 43.9±2.5°, HA-CNT: 39.7±4.3°) and increased local modulus that was measured through atomic force microscopy (HA: 74.93±12.6 kPa, HA-CNT:174.85±31.9 kPa). Bulk mechanical properties, measured through tensile testing was not significantly different between the two scaffolds (HA: 1.417±0.26 MPa, HA-CNT:1.769±0.26 MPa) [17].

We found that both WT and NF SC show increased proliferation on aligned fibers compared to hydrogel samples. Aspect ratio was higher for NF SC than WT SC and increased with the inclusion of a collagen coating. The NF and WT SCs cultured on collagen coated nanofibers interact with the fibers through integrin receptors on their cell membranes emulating the interaction of cell-ECM interaction in physiology. Therefore, adsorbed collagen coating present on the fibers enhance cell adhesion, spreading and alignment on HA and HA-CNT fibers. Cell spread area was higher for WT SC, while aspect ratio was higher for NF SC indicating that the NF cells have a more spindle shaped morphology. Our previous research with DRG neurons on HA-CNT nanofibers showed complementary results [17]. HA-CNT showed increased neurite outgrowth compared to HA nanofiber scaffolds. We attributed these results to increased fiber roughness of HA-CNT which can be seen in the SEM images (Fig 2). The increased conductivity of HA-CNT would also affect cellular behavior and communication between cells, which could contribute to increased neurite growth and elongation of SC. Finding significant differences in cell morphology between WT and NF SC can be attributed to NF cells having a more migratory phenotype in vivo under no additional stimulation (S5 Fig in S1 File).

Next, we evaluated electrical stimulation of cells using our custom fabricated stimulation well plates. Electrical conductivity of the HA-CNT material was evaluated and published in our previous work through impedance spectroscopy and cyclic voltammetry [17]. These results showed that 0.01% CNT decreased impedance and increased the charge storage capacitance of the material over HA nanofibers alone. We evaluated 100 and 200 mV/mm stimulation conditions to match our previous work, with 100 being withing the typical range of stimulation during regeneration and 200 being higher. DRG neuron studies showed increased neurite outgrowth at 100mV/mm stimulation on our materials [17]. In WT SC, we see that stimulation in the range we tested (0–200 mV/mm) increased SC elongation, decreased cell area (roundness) and increased proliferation. This matched published research by Koppes et al. group conducted stimulation study on Schwann cells on thinly coated laminin with a different stimulatory model [33]. Their results also indicated that at a voltage high as 200mV the cell viability decrease compared to lower voltages.

It is known that endogenous (21–140 mV/mm) electrical fields are present during development and following injury [23]. Neuron hyperexcitability has been shown to influence SC behaviors including proliferation, differentiation and myelination [33]. In vivo, we know that SC experience stimulation from neurons. The previously published work on neurons and WT SC shows pro-regenerative qualities under the stimulation in the range of endogenous electric fields present during injury. This provides evidence that our materials may be useful in tissue regeneration paradigms where increased neurite outgrowth and increased SC proliferation may be important for reconnection of severed neurites.

For NF SC, we saw different results in the same stimulation range. Elongation and aspect ratio decreased, while proliferation stayed about constant. This could be attributed to differences in electrical stimulation in the regeneration vs disease states. Electric fields are continually present in healthy neurons, though this is at a significantly lower potential(2–6 mV/mm), then following injury [33]. It is known that SC proliferate during NF tumor formation and growth as these cells are contributors to the tumor mass, along with fibroblasts, endothelial cells and immune cells. It appears that these cells have lost their ability to respond appropriately to higher ES parameters in terms of regulation of cell proliferation and NGF secretion. These cells proliferate more on conductive scaffolds without stimulation that may better mimic the tumor microenvironment. Future work will investigate NF SC at very low voltages to determine if NF SC respond differently in these parameters.

We then investigated NGF release from WT and NFSC cells under the stimulation conditions. Again, we find increased NGF release from WT SC at the stimulation conditions with the highest release at 100 mV/mm. NF SC show relatively stable NGF release in all conditions. This could attribute to WT SC being stimulated at regeneration ranges which triggers them to release NGF to support regeneration. Interestingly, our previous DRG studies also showed optimal neurite outgrowth at 100 mV/mm with lower growth at 200 mV/mm. Neurons influence NGF production in SC and this work shows that this could partially be due the electrical stimulation (from underlying neurons).

WT response to ES releasing NGF was compared and confirmed with other studies. The study by Koppes et al. confirmed the release of NGF mediated through ES by healthy Schwann cells. Their research confirmed that DRG neurons cultured on conditioned media extracted from stimulated Schwann cell has the ability to enhance the neurite extension [33, 34]. In addition, results of Wang et al. study also confirm the ability of Schwann cells to release NGF when cultured on reduced graphene oxide (RGO) scaffolds [35]. NGF ELISA was conducted on the NF Schwann cells to compare and confirm the degree of cellular behavior from healthy/ WT cells.

Understanding the gene expression changes in NF1 is complex as these tumors often contain several cell types including neurons, SC, fibroblasts, and immune cells, all of which are communicating with each other. A recent paper has used single-cell RNA sequencing (porcine model) to look at the various cell types can give us clues on potential changes in gene expression between wild-type and NF1 SC [36]. This study revealed that cells in the neurofibroma are similar to those in a regenerative/immunosuppression state, which are likely signaled by the NF SC. NF1 tumor formation is initiated by the loss of contact between the SC and the axon beginning the process of tumor formation [37]. Taken in this context, the differences between WT SC and NF SC behavior on the conductive nanofibers found in this study could provide another avenue to investigate. Gene expression changes in SC could be signaled by stimulation from underlying neurons. In wild-type SC, SC polarization, growth factor release and migration have been shown to be affected by electrical stimulation [18]. Our future work will look at gene expression in WT SC and NF SC under stimulatory conditions, focusing on SOX10, OCT16, GFAP and MBP, which are genes that play a role in SC maturity and proliferation [38]. Co-culture studies with other cells in the tumor environment will also be critical to understanding this disease state.

Taken together, these results have major implications for SC behavior following electrical stimulation. Firstly, in the regeneration scenario, including an electrically conductive material and stimulation has positive effects on SC behavior including increased proliferation, elongation and NGF release. Our published data on neurons showed HA-CNT and 100 mV/mm stimulation is promising for nerve regeneration applications. In the NF disease state, SCs behave differently. Understanding SC behavior is critical for understanding neurofibromatosis tumor formation and growth as these tumors are initiated by SC proliferation. We found that topographical cues or inherent electrical conductivity (at low voltages) could increase cell proliferation (Fig 4). Following stimulation at 100 mV/mm and higher, these SC exhibit abnormal clustering, decreases in elongation and stagnation in proliferation. This has two major conclusions. 1. Electrical stimulation seems to halt SC proliferation and could be used in therapeutic applications. 2. Understanding the differences between WT and NF SC is important for understanding the disease state and therefore, it would be interesting to investigate if there are lower endogenous levels of stimulation that could lead to hyperproliferation in NF SC. We will use these results to further develop our in vitro organotypic model of NF1.

## Conclusion

In conclusion we have demonstrated a platform that can be utilized for in vitro testing of WT and NF Schwann cells. WT Schwann cells cultured on this multiple cue model demonstrate positive characteristics useful in peripheral nerve regeneration applications. The study concluded that NF Schwann cells have lost the ability to respond in a pro-regenerative manner through morphological changes and control of proliferation and NGF secretion when exposed to high exogenous electrical stimulation. However on electroconductive nanofibers, they show increased elongation and proliferation, concluding that this may better mimic the tumor microenvironment. Our future work will investigate SC behavior at low electrical stimulation and co-culture of NF SC with other cells in the tumor microenvironment to develop and organotypic model for drug testing.

## Supporting information

S1 File Five supplementary figures are included in the supplementary file that include NMR, TEM, Fig 3 individual channels, Fig 5 individual channels and an image of overlayed nanofibers. (DOCX)

## References

[1] Ferrer M., et al., Pharmacological and genomic profiling of neurofibromatosis type 1 plexiform neurofibroma-derived schwann cells. Scientific Data, 2018. 5(1): p. 180106. doi: 10.1038/sdata.2018.106 29893754 PMC5996849

[2] Prada C.E., et al., Pediatric plexiform neurofibromas: impact on morbidity and mortality in neurofibromatosis type 1. J Pediatr, 2012. 160(3): p. 461–7. doi: 10.1016/j.jpeds.2011.08.051 21996156

[3] Carrió M., et al., Reprogramming Captures the Genetic and Tumorigenic Properties of Neurofibromatosis Type 1 Plexiform Neurofibromas. Stem Cell Reports, 2019. 12(2): p. 411–426. doi: 10.1016/j.stemcr.2019.01.001 30713041 PMC6373434

[4] Kraniak J.M., et al., Development of 3D culture models of plexiform neurofibroma and initial application for phenotypic characterization and drug screening. Experimental neurology, 2018. 299(Pt B): p. 289–298. doi: 10.1016/j.expneurol.2017.10.012 29055717 PMC6863155

[5] Daston M.M., et al., The protein product of the neurofibromatosis type 1 gene is expressed at highest abundance in neurons, Schwann cells, and oligodendrocytes. Neuron, 1992. 8(3): p. 415–28. doi: 10.1016/0896-6273(92)90270-n 1550670

[6] Kluwe L., Friedrich R., and Mautner V.-F., Loss of NF1 allele in schwann cells but not in fibroblasts derived from an NF1-associated neurofibroma. Genes, Chromosomes and Cancer, 1999. 24(3): p. 283–285. doi: 10.1002/(sici)1098-2264(199903)24:3&lt;283::aid-gcc15&gt;3.0.co;2-k 10451710

[7] Le L.Q. and Parada L.F., Tumor microenvironment and neurofibromatosis type I: connecting the GAPs. Oncogene, 2007. 26(32): p. 4609–4616. doi: 10.1038/sj.onc.1210261 17297459 PMC2760340

[8] Gutmann D.H., et al., Modulation of the neurofibromatosis type 1 gene product, neurofibromin, during Schwann cell differentiation. Journal of Neuroscience Research, 1993. 36(2): p. 216–223. doi: 10.1002/jnr.490360212 7505343

[9] Sheela S., Riccardi V.M., and Ratner N., Angiogenic and invasive properties of neurofibroma Schwann cells. Journal of Cell Biology, 1990. 111(2): p. 645–653. doi: 10.1083/jcb.111.2.645 1696266 PMC2116200

[10] Mazuelas H., et al., Modeling iPSC-derived human neurofibroma-like tumors in mice uncovers the heterogeneity of Schwann cells within plexiform neurofibromas. Cell Reports, 2022. 38(7): p. 110385. doi: 10.1016/j.celrep.2022.110385 35172160

[11] Dombi E., et al., Activity of Selumetinib in Neurofibromatosis Type 1-Related Plexiform Neurofibromas. N Engl J Med, 2016. 375(26): p. 2550–2560. doi: 10.1056/NEJMoa1605943 28029918 PMC5508592

[12] Gross A.M., et al., Selumetinib in Children with Inoperable Plexiform Neurofibromas. New England Journal of Medicine, 2020. 382(15): p. 1430–1442. doi: 10.1056/NEJMoa1912735 32187457 PMC7305659

[13] Chen Z., et al., Spatiotemporal Loss of NF1 in Schwann Cell Lineage Leads to Different Types of Cutaneous Neurofibroma Susceptible to Modification by the Hippo Pathway. Cancer Discovery, 2019. 9(1): p. 114–129. doi: 10.1158/2159-8290.CD-18-0151 30348677 PMC6328325

[14] Edmondson R., et al., Three-Dimensional Cell Culture Systems and Their Applications in Drug Discovery and Cell-Based Biosensors. ASSAY and Drug Development Technologies, 2014. 12(4): p. 207–218. doi: 10.1089/adt.2014.573 24831787 PMC4026212

[15] Mazuelas H., Carrio M., and Serra E., Modeling tumors of the peripheral nervous system associated with Neurofibromatosis type 1: Reprogramming plexiform neurofibroma cells. Stem Cell Res, 2020. 49: p. 102068. doi: 10.1016/j.scr.2020.102068 33160273

[16] Gutmann D.H. and Giovannini M., Mouse models of neurofibromatosis 1 and 2. Neoplasia, 2002. 4(4): p. 279–90. doi: 10.1038/sj.neo.7900249 12082543 PMC1531708

[17] Steel E.M., Azar J., and Sundararaghavan H.G., Electrospun hyaluronic acid-carbon nanotube nanofibers for neural engineering Materialia, 2020. 9.

[18] Hu M., et al., Electrical stimulation enhances neuronal cell activity mediated by Schwann cell derived exosomes. Sci Rep, 2019. 9(1): p. 4206. doi: 10.1038/s41598-019-41007-5 30862846 PMC6414536

[19] Wenjin W., et al., Electrical stimulation promotes BDNF expression in spinal cord neurons through Ca(2+)- and Erk-dependent signaling pathways. Cell Mol Neurobiol, 2011. 31(3): p. 459–67. doi: 10.1007/s10571-010-9639-0 21259048 PMC11498367

[20] Shi G., Zhang Z., and Rouabhia M., The regulation of cell functions electrically using biodegradable polypyrrole–polylactide conductors. Biomaterials, 2008. 29(28): p. 3792–3798. doi: 10.1016/j.biomaterials.2008.06.010 18602689

[21] Gartner A. and Staiger V., Neurotrophin secretion from hippocampal neurons evoked by long-term-potentiation-inducing electrical stimulation patterns. 2002. 99(9): p. 6386–6391. doi: 10.1073/pnas.092129699 11983920 PMC122958

[22] Brighton C.T., The Effect of Electrical Fields on Gene and Protein Expression in Human Osteoarthritic Cartilage Explants. 2008. 90(4): p. 833. 10.2106/JBJS.F.01437 18381322

[23] Nuccitelli R., Endogenous electric fields in embryos during development, regeneration and wound healing. Radiat Prot Dosimetry, 2003. 106(4): p. 375–83. doi: 10.1093/oxfordjournals.rpd.a006375 14690282

[24] Caño Silva V. and Serrano Afonso A., Neuropathic Pain due to Neurofibromatosis Treated With Transcutaneous Electrical Nerve Stimulation in a Pregnant Patient: A Case Report. A&A Practice, 2019. 13(9): p. 329–331. 31361663 10.1213/XAA.0000000000001068 PMC6818987

[25] Sundararaghavan H.G., Metter R.B., and Burdick J.A., Electrospun fibrous scaffolds with multiscale and photopatterned porosity. Macromol Biosci, 2010. 10(3): p. 265–70. doi: 10.1002/mabi.200900363 20014198 PMC3021958

[26] Mays E.A., et al., Enzyme-Mediated Nerve Growth Factor Release from Nanofibers Using Gelatin Microspheres. Tissue Eng Part A, 2023. 29(11–12): p. 333–343. doi: 10.1089/ten.TEA.2022.0205 37016821

[27] Li H., et al., Immortalization of human normal and NF1 neurofibroma Schwann cells. Laboratory Investigation, 2016. 96(10): p. 1105–1115. doi: 10.1038/labinvest.2016.88 27617404

[28] Whitehead T.J., Avila C.O.C., and Sundararaghavan H.G., Combining growth factor releasing microspheres within aligned nanofibers enhances neurite outgrowth. J Biomed Mater Res A, 2018. 106(1): p. 17–25. doi: 10.1002/jbm.a.36204 28879680

[29] Wrobel M.R. and Sundararaghavan H.G., Directed migration in neural tissue engineering. Tissue Eng Part B Rev, 2014. 20(2): p. 93–105. doi: 10.1089/ten.TEB.2013.0233 23815309

[30] Wrobel M.R. and Sundararaghavan H.G., Positive and negative cues for modulating neurite dynamics and receptor expression. Biomed Mater, 2017. 12(2): p. 025016. doi: 10.1088/1748-605X/aa61d1 28221164

[31] Sundararaghavan H.G. and Burdick J.A., Gradients with depth in electrospun fibrous scaffolds for directed cell behavior. Biomacromolecules, 2011. 12(6): p. 2344–50. doi: 10.1021/bm200415g 21528921 PMC3115661

[32] Sundararaghavan H.G., et al., Fiber alignment directs cell motility over chemotactic gradients. Biotechnol Bioeng, 2013. 110(4): p. 1249–54. doi: 10.1002/bit.24788 23172355

[33] Koppes A.N., et al., Electrical stimulation of schwann cells promotes sustained increases in neurite outgrowth. Tissue Eng Part A, 2014. 20(3–4): p. 494–506. doi: 10.1089/ten.TEA.2013.0012 24063574 PMC3926181

[34] Koppes A.N., et al., Neurite outgrowth on electrospun PLLA fibers is enhanced by exogenous electrical stimulation. J Neural Eng, 2014. 11(4): p. 046002. doi: 10.1088/1741-2560/11/4/046002 24891494 PMC4873603

[35] Wang J., et al., In vitro and in vivo studies of electroactive reduced graphene oxide-modified nanofiber scaffolds for peripheral nerve regeneration. Acta Biomater, 2019. 84: p. 98–113. doi: 10.1016/j.actbio.2018.11.032 30471474

[36] McLean D.T., et al., Single-cell RNA sequencing of neurofibromas reveals a tumor microenvironment favorable for neural regeneration and immune suppression in a neurofibromatosis type 1 porcine model. Front Oncol, 2023. 13: p. 1253659. doi: 10.3389/fonc.2023.1253659 37817770 PMC10561395

[37] Ribeiro S., et al., Injury signals cooperate with Nf1 loss to relieve the tumor-suppressive environment of adult peripheral nerve. Cell Rep, 2013. 5(1): p. 126–36. doi: 10.1016/j.celrep.2013.08.033 24075988

[38] Jessen K.R. and Mirsky R., The origin and development of glial cells in peripheral nerves. Nat Rev Neurosci, 2005. 6(9): p. 671–82. doi: 10.1038/nrn1746 16136171

