# Supplementary material for: Electrical stimulation of Schwann cells on electrospun hyaluronic acid carbon nanotube fibers
Source: PLoS One. 2024 Aug 7;19(8):e0308207. doi: 10.1371/journal.pone.0308207 (PMC11305570; doi:10.1371/journal.pone.0308207)
Supplement: S1 File — (DOCX) [file pone.0308207.s001.docx]

**Supplementary files:**

Electrical Stimulation of Schwann Cells on Electrospun Hyaluronic Acid Carbon Nanotube Fibers

Judy Senanayake^1^, Raymond R. Mattingly^2^, Harini G. Sundararaghavan^1^

Supplemental Figure 1

**
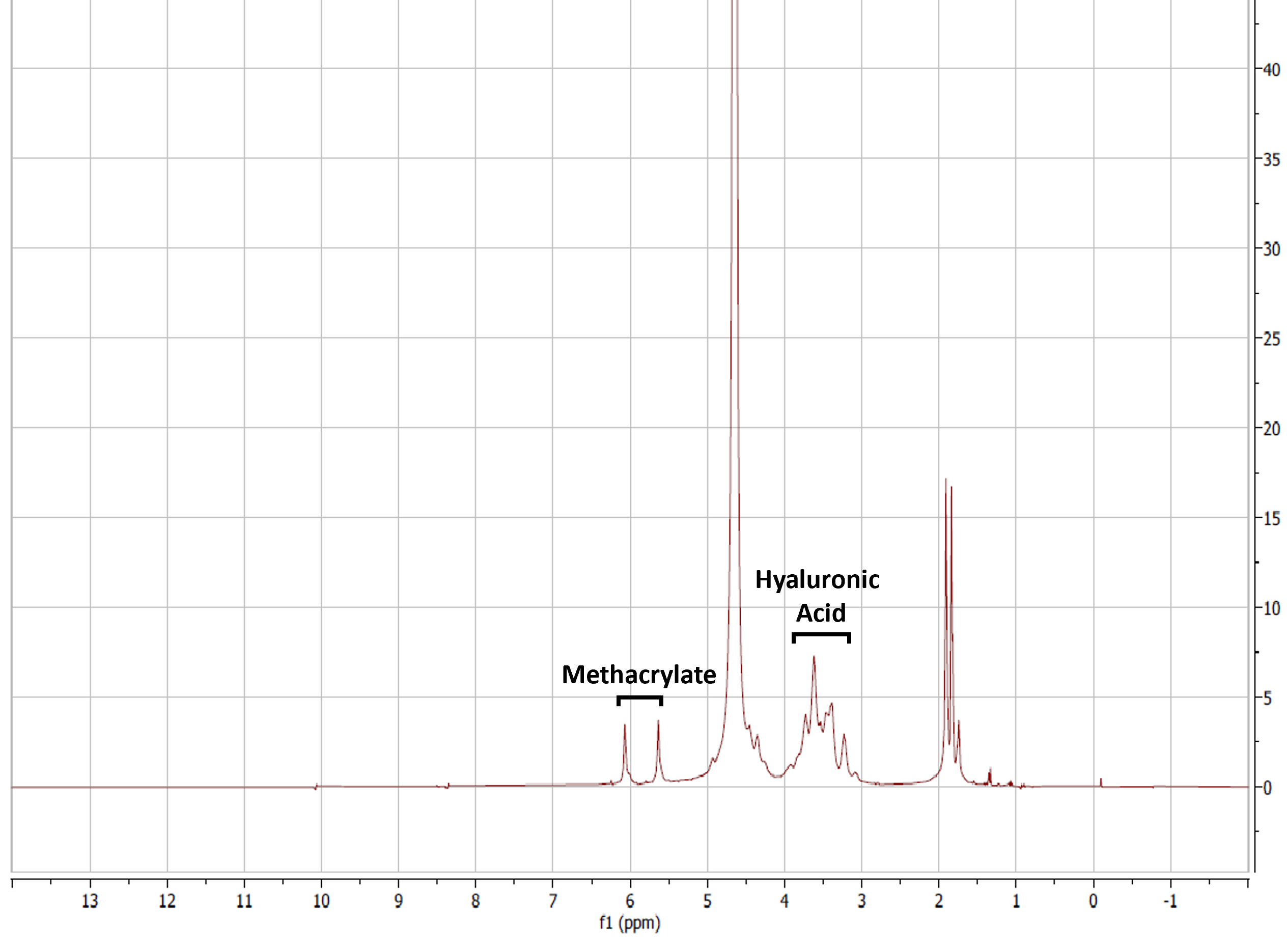
**

**Supplemental Figure 1:** Sample NMR graph of Methacrylated Hyaluronic Acid. Methacrylate percentage is calculated using a ratio of the area under the methacrylate peak and the area under the hyaluronic acid peak.

Supplemental Figure 2


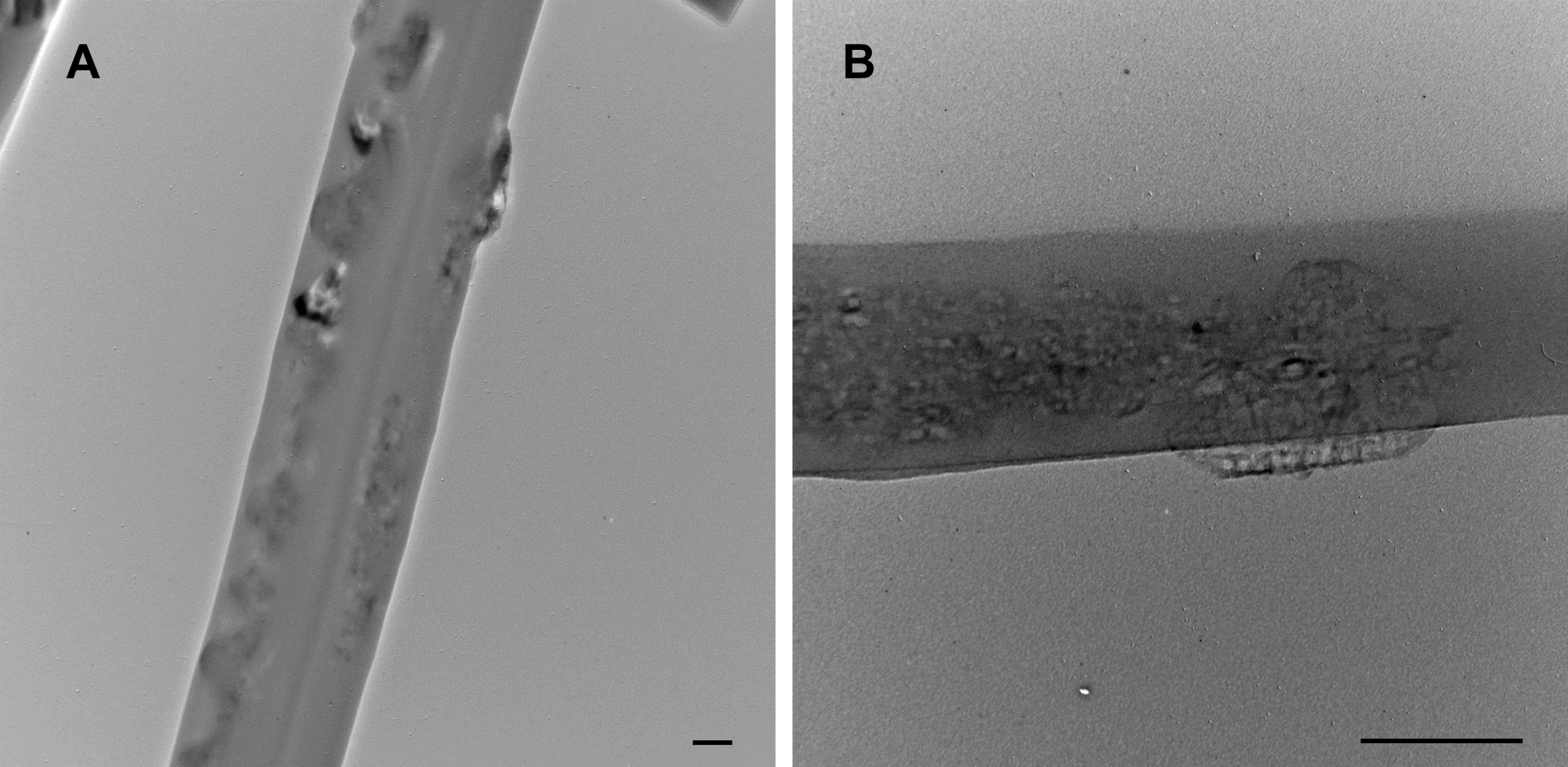


**Supplemental Figure 2:** Sample TEM images of carbon nanotubes within hyaluronic acid nanofibers. (A,B) Carbon nanotubes are imbedded in the HA nanofiber. CNT protrusions can be seen on nanofibers using TEM. These bumps are in the same size order as the roughness on SEM images of HA-CNT nanofibers. Scale bar = 100 nm.

Supplemental Figure 3


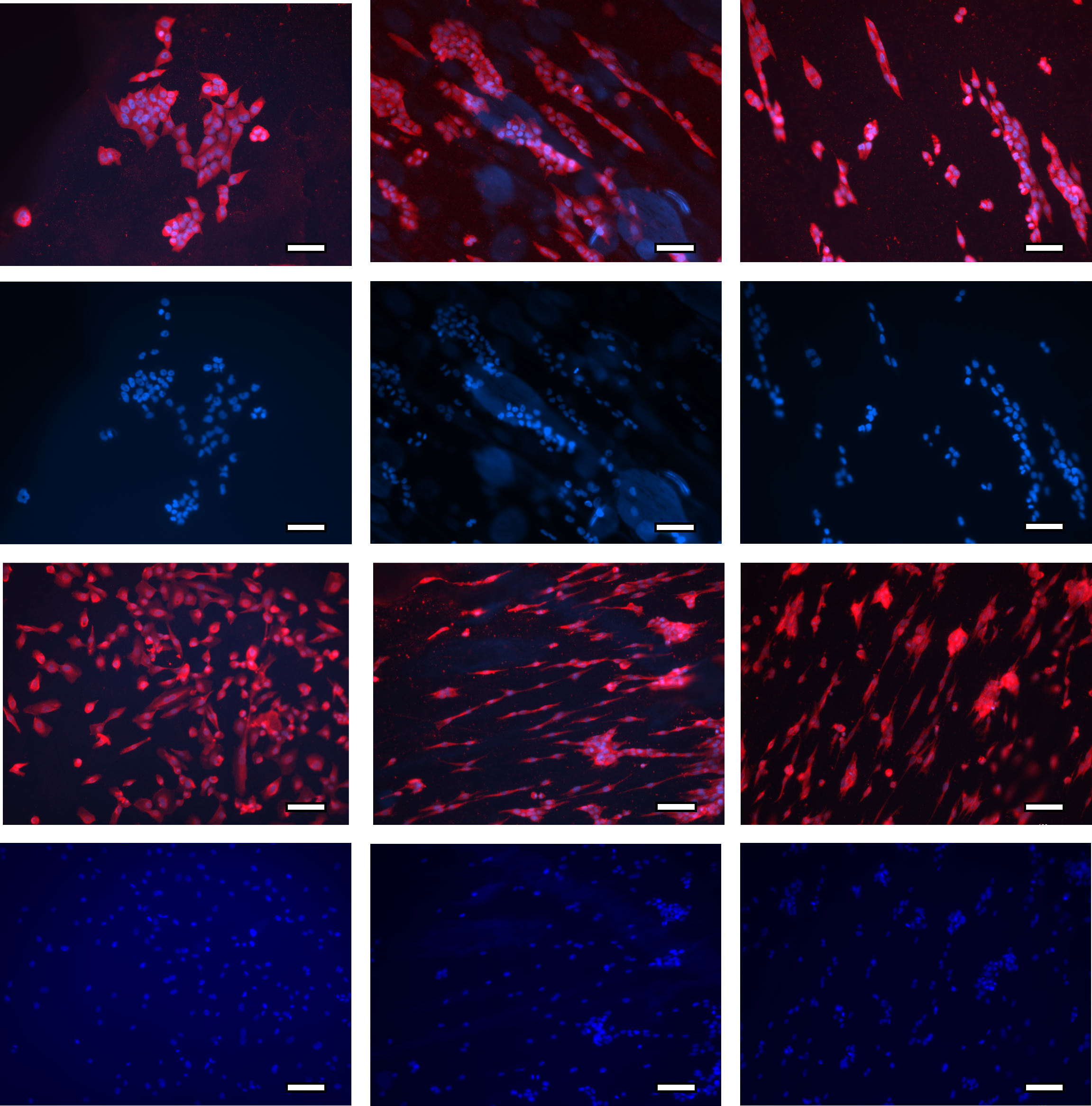


Supplemental Figure 3: Individual s100b and DAPI channels that correspond to Figure 3 in the main paper. Both channels can be visualized. SB=100µm

Supplemental Figure 4


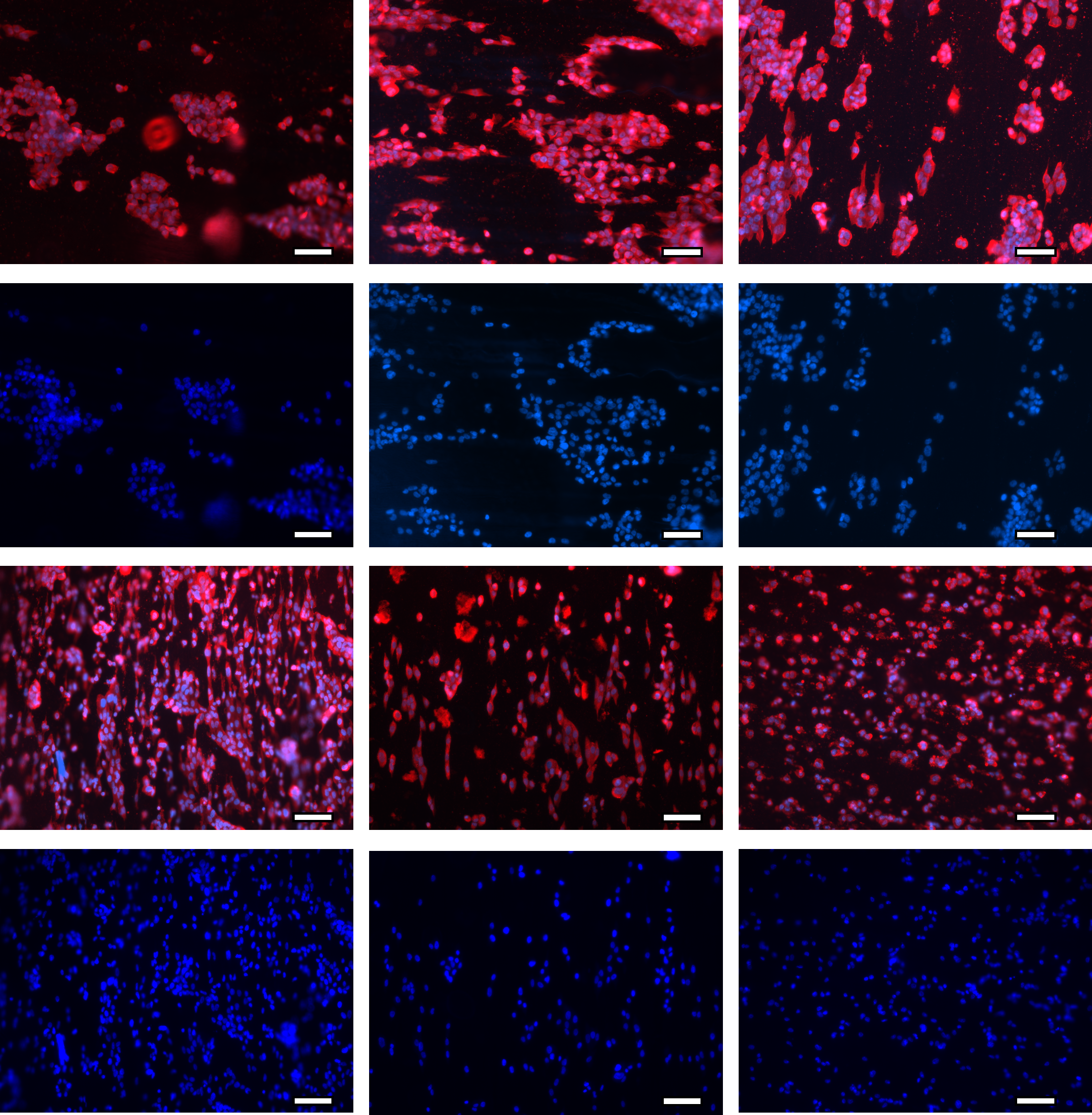


Supplemental Figure 4: Individual s100b and DAPI channels that correspond to Figure 5 in the main paper. Both channels can be visualized. SB=100µm

Supplemental Figure 5

Supplemental Figure 5: Overlayed images showing SC (S100b, red), cell nuclei (DAPI, light blue) and brightfield image of HA-CNT nanofibers. (A) WT SC, (B) NF SC. Scale bar = 50 µm. White arrow indicates fiber direction.
